# Rabies Virus-Induced Autophagy Is Dependent on Viral Load in BV2 Cells

**DOI:** 10.3389/fmicb.2021.595678

**Published:** 2021-05-25

**Authors:** Yang Wang, Hongling He, Jiesen Li, Luman Chen, Jun Luo, Yanqi Kuang, Ziyu Lv, Ruqi Fan, Boyue Zhang, Yongwen Luo, Xiaofeng Guo

**Affiliations:** College of Veterinary Medicine, South China Agricultural University, Guangzhou, China

**Keywords:** rabies virus, BV2, autophagy, autophagosomes, viral load

## Abstract

An increasing number of studies are showing that autophagy plays a vital role in viral replication and escape. Rabies virus (RABV), a typical neurotropic virus, has been proven to induce autophagy in neurons. However, there are no reports indicating that RABV can cause autophagy in other cells of the central nervous system. Thus, we aimed to explore the relationship between autophagy and RABV infection in BV2 cells in this study. Results of viral growth curves showed that the titers of microglial BV2 cells infected with RABV peaked at 12 hours post-infection (hpi) and then decreased continuously over time. However, it was found that the viral genome RNA and structural proteins can express normally in BV2 cells. In addition, Western blotting indicated that RABV infection increased LC3-II and p62 expression in BV2 cells. LC3 punctate increased with RABV infection in BV2 cells after the transfection of fluorescent protein-tagged LC3 plasmids. Moreover, autophagy cargo protein further accumulated with RABV infection in Bafilomycin A1-treated cells. Subsequently, RABV infection inhibited the fusion of autophagosomes with lysosomes by using a tandem fluorescent marker. Furthermore, a higher multiplicity of infection induced stronger autophagy. Thus, RABV can induce autophagy in BV2 cells, and the autophagy is positively associated with the viral load.

## Introduction

Rabies virus (RABV), belonging to the *Rhabdoviridae* family—*Lyssavirus* genus, is an unsegmented negative-stranded RNA virus, which causes a zoonotic infectious disease. RABV usually invades the central nervous system (CNS), causing serious encephalitis and neurological symptoms, ultimately leading to irreversible damage and consequent death (Fooks et al., 2017). Currently, the only effective treatment against RABV is vaccination and anti-RABV immunoglobulin administration before or after exposure.

Autophagy is a highly conserved process of cell degradation and recycling in all eukaryotes (Parzych and Klionsky, 2014), which can defend against microbial invasion by activating innate and adaptive immunity or by direct capture and degradation (Sharma et al., 2018). At the same time, viruses have evolved to destroy or use autophagy to benefit themselves (Choi et al., 2018). Thus, autophagy can be a treatment target. Autophagy plays a dual role in promoting and resisting viruses during Zika virus replication (Chiramel and Best, 2018). Autophagy can promote the presentation of measles virus epitopes on MHC-II molecules and subsequent initiation of CD4^+^ T-cell response by the adaptive immune system (Rozieres et al., 2017). Studies have shown that autophagy induced by HIV depends on cell-type specificity (Espert et al., 2009). Autophagy has an anti-HIV effect by inducing selective degradation of the viral transactivator Tat in CD4^+^ T lymphocytes (Sagnier et al., 2015). Hepatitis C virus (HCV) induces autophagy *via* endoplasmic reticulum stress and unfolded protein responses (Lee et al., 2015; Chusri et al., 2016). Our previous study found that the wild-type RABV induces autophagy in mouse neuroblastoma (NA) and human neuroblastoma (SK) cells, but the autophagic flux suggests the contrary (Peng et al., 2016). Another study proved that the RABV phosphoprotein interacts with BECN1 to inhibit CASP2 expression and activates the downstream AMPK-MAPK/AKT-MTOR pathway cascade to induce endogenous autophagy in NA cells (Liu et al., 2017). The previous study has indicated that natural compounds inhibit RABV replication by inhibiting autophagy in baby hamster kidney cells (Tu et al., 2018). Therefore, autophagy is a potential target for inhibiting RABV replication. However, few studies have investigated RABV-induced autophagy in other cells of the CNS except neuroblastoma.

Microglia, the resident immune cells of the CNS, not only play the role of immune surveillance and immune clearance by participating in pro-inflammatory responses in the brain but also play essential roles in growth and injury protection in the CNS (Orihuela et al., 2016). Remarkably, microglia can be infected by RABV (Ray et al., 1997). BV2 cells are immortalized cells obtained by infecting murine microglia with a v-raf/v-myc oncogene-carrying retrovirus (Blasi et al., 1990). A previous study has shown that autophagy in BV2 cells can negatively regulate the release of pro-inflammatory factors such as NO, IL-6, IL-1β, and TNF-α (Bussi et al., 2017). Further, on pharmacological intervention to inhibit autophagy, increased inflammation is observed (Li et al., 2019). Because, at the end of the course of rabies, death is often caused by severe encephalitis, we aimed to explore whether RABV can induce autophagy in BV2 cells to further identify the relationship between autophagy and the pathogenicity of RABV.

## Materials and Methods

### Cells, Viruses, Antibodies, and Plasmids

BV2 cells (Wuhan Institute of Biological Products, Wuhan, China), a murine microglial cell line, were cultured in Dulbecco’s modified Eagle’s medium (DMEM; Gibco, Grand Island, NY, United States) supplemented with 10% fetal bovine serum (FBS; Gibco). Mouse neuroblastoma (NA) cells (Wuhan Institute of Biological Products) were maintained in RPMI 1640 (Gibco) containing 10% FBS. RABV strains HEP-Flury (vaccine strain) and CVS-11 (virulent strain) were obtained from the Department of Microbiology and Immunology, School of Veterinary Medicine, South China Agricultural University (Guangzhou, China). Rabbit anti-LC3B antibody (#3868) was purchased from Cell Signaling Technology (Boston, MA, United States). Rabbit anti-p62 antibody (A0682) was purchased from ABclonal (Wuhan, China). Mouse anti-NBR1 antibody (sc-130380) was purchased from Santa Cruz Biotechnology (Dallas, TX, United States). Mouse anti-β-actin antibody (AA128) was purchased from Beyotime (Shanghai, China). Fluorescein isothiocyanate (FITC)-labeled anti-RABV-N antibodies were obtained from Fujirebio Inc. (Malvern, PA, United States). Anti-RABV-P antibody was purchased from Zhejiang Tongdian Biotechnology Co., Ltd. (Zhejiang, China). mRFP-EGFP-LC3 (#21074) was purchased from Addgene (Cambridge, MA, United States).

### Viral Propagation and Titration

CVS-11 and HEP-Flury strains were propagated in NA cells. Viral titrations were performed by direct fluorescent antibody (dFA) assay as previously described (Luo et al., 2020). In brief, NA cells grown in 96-well cell culture plates were inoculated with 10-fold serial dilutions of the indicated virus in RPMI 1640 medium and incubated at 37°C for 2 days. The culture medium was discarded, and the cells were fixed with 80% acetone for 30 min at −20°C. The cells were then washed in PBS and stained with FITC-labeled anti-RABV-N antibodies (1:500) overnight at 4°C. Antigen-positive foci were photographed and counted under a fluorescence microscope (AMG, Mill Creek, WA, United States; focus forming units per milliliter, FFU/ml).

### Virus Growth Assay

Monolayers of BV2 cells cultured at 37°C in 100 mm cell culture plates were infected with RABV at a multiplicity of infection (MOI) of 1. The cultured supernatants were collected at 12, 24, 36, and 48 hpi. Viral titers were determined in NA cells by dFA as described above.

### Western Blotting

BV2 cells infected with RABV were lysed at indicated time points. The lysate protein was quantified by spectrophotometry (NanoDrop 2000c, Thermo Fisher Scientific, Waltham, MA, United States). An equal amount of each sample was separated *via* 12.5% sodium dodecyl sulfate-polyacrylamide gel electrophoresis and transferred onto polyvinylidene fluoride membranes (Sigma-Aldrich, 3010040001). After blocking with 5% nonfat dry milk containing 0.05% Tween 20 for 1 h at room temperature, the membranes were incubated overnight with primary antibodies (1:1,000) first at 4°C, followed by incubation with secondary antibodies (Beyotime, A0208/A0216; 1:50,000) conjugated with horseradish peroxidase at 37°C for 1 h. The immunoreactive bands were visualized by enhanced chemiluminescence on the Fine-do X6 system (Tanon, Shanghai, China). The relative amount of the target protein was normalized to the amount of β-actin. The intensity of protein bands was quantified using the ImageJ software (National Institutes of Health, Bethesda, MD, United States).

### Cell Transfection

Monolayers of BV2 cells cultured at 37°C in 20 mm special cell culture plates were transfected with Lipofectamine 3000 (Thermo Fisher Scientific) according to the manufacturer’s instructions. After transfection for 12 h, the cells were inoculated with RABV at an MOI of 3. A laser confocal fluorescence microscope (Leica, Wetzlar, Germany) was used to observe the positive fluorescence spots in cells at indicated time points.

### Reverse Transcription Quantitative Polymerase Chain Reaction

BV2 cells infected with RABV were harvested using TRIzol reagent (Magen, Guangzhou, China) according to the manufacturer’s instructions at indicated time points. Reverse transcription was performed using the HiScript II First Strand cDNA Synthesis Kit (Vazyme, Nanjing, China). Each sample was tested in triplicate using the SYBR Green Master Mix (Vazyme). Reverse Transcription Quantitative Polymerase Chain Reaction (RT-qPCR) was performed using a CFX connect real-time system (Bio-Rad, Hercules, CA, United States). The levels of N mRNA, P mRNA, M mRNA, G mRNA, L mRNA, and genome RNA (gRNA) were normalized using glyceraldehyde-3-phosphate dehydrogenase (GAPDH). Primers are shown at the bottom of this article (**Table 1**).

**Table 1:** Primers used for the analysis of genes expression.

Gene	Forward primer (5'-3')	Reverse primer (5'-3')
CVS-11 N	TCACCGCAAGGGAAGCA	AACGGAAGTGGATGAAATAAGAG
CVS-11 P	CGTACCTGGATAATGTTGGAGTC	TTGGAGGGTTAGGAAAGTTGA
CVS-11 M	CTCCGTATGACGATGACCTG	CATTTGGGCTACACGCTTT
CVS-11 G	ATGCCCGAGAATCCGAGAC	CATCCACAAAGCCGCAAGT
CVS-11 L	CCCTCATTACCTACCAGTCTCA	ACCCAGCACTTGCCTCAT
CVS-11 genome	AGAAGAAAAAGACAGCGTCAATTG	AGAGACCACCTGATTATTGACTTTGA
HEP-Flury N	TTTAGTCGGTCTTCTCCTGAGTCT	AATCTGCTCTATTCTATCCGCAATGT
HEP-Flury P	GAGTCCAAATAGTCAGACAAATGAGGT	AGGAAAGTTGACCGAGACATAGGA
HEP-Flury M	AGAGGACAAAGACTCTTCTCTGCT	TGGAGTTAAGCCCGTATGTTCTCT
HEP-Flury G	GCCTTGATTGCCCTGATGTTGATAA	CATTTCTCCCTGTCCCTCCAAGAT
HEP-Flury L	TGTTGATGTCTGATTTCGCATTGTCT	AAGGGAACGCTCTTGACAGATGT
HEP-Flury genome	AGAAGAAGCAGACATCGTCAGTTG	GGAGACCACCTGATTATTGACTTTGA
GAPDH	CGTCCCGTAGACAAAATGGT	TTGATGGCAACAATCTCCAC

### Statistical Analysis

Values are presented as mean ± standard deviation (SD). Three independent experiments were performed for all parameters. Data were analyzed using GraphPad Prism 6 software (GraphPad Software, San Diego, CA, United States). Statistically significant differences were determined *via* Student’s *t-*test. *p* < 0.05 was considered as statistically significant.

## Results

### The Production of RABV Progeny Particles Was Inhibited in BV2 Cells

**Figure 1:**
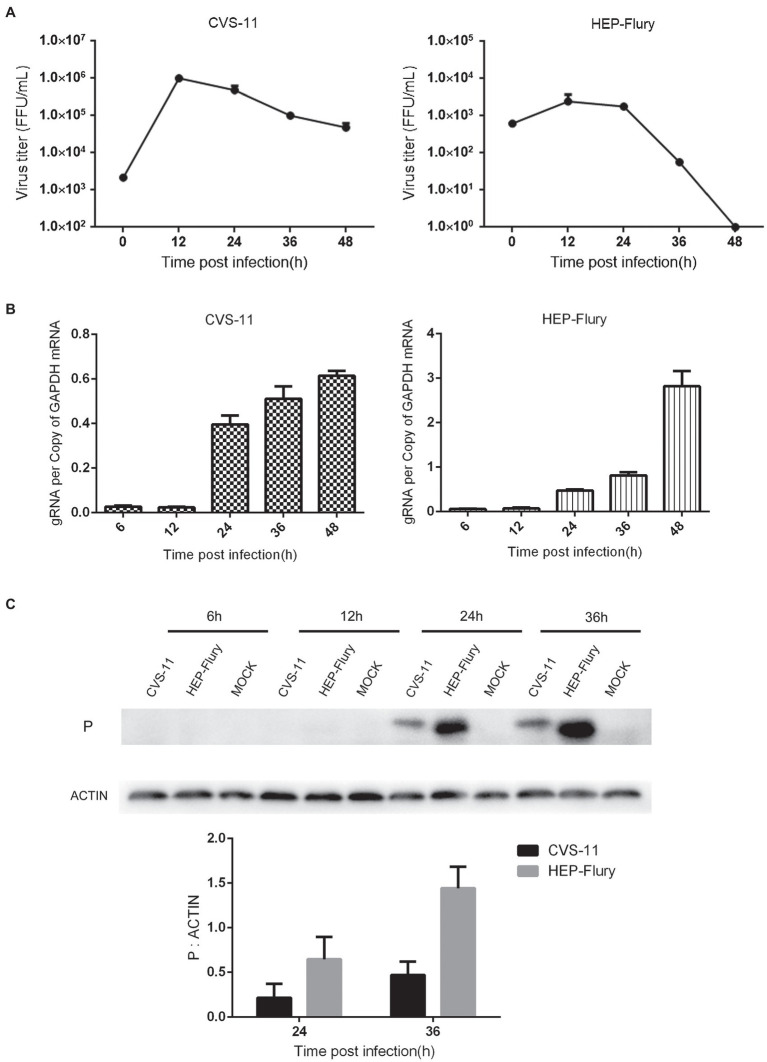
Growth characteristics of the RABV on BV2 cells. **(A)** Growth curves of RABV in BV2 cells. BV2 cells were infected with CVS-11 or HEP-Flury at an MOI of 1 and continuously cultured at 37°C. Cultured supernatants were collected at 12, 24, 36, and 48 hpi. Then, viral titrations were performed by direct fluorescent antibody assay. Virus titers were assayed in triplicate. Data are shown as mean ± SD. **(B)** Detection of RABV gRNA by RT-qPCR. BV2 cells were, respectively, infected with CVS-11 or HEP-Flury at an MOI of 1. Cells were harvested at 6, 12, 24, 36, and 48 hpi. The levels of gRNA in BV2 cells were determined by RT-qPCR using a CFX connect real-time system. The relative gRNA levels were normalized to GAPDH. Data are presented as mean ± SD, *n* = 3. **(C)** Detection of structural protein expression by Western blot. BV2 cells were, respectively, infected with CVS-11 or HEP-Flury at an MOI of 1. Cell lysates were harvested at 6, 12, 24, and 36 hpi for Western blot analysis. The results present the relative expression levels of rabies phosphoprotein and β-actin visualized using the ImageJ software. Data are shown as mean ± SD, *n* = 3.

Viral growth curves were determined in BV2 cells to investigate the growth characteristics. As shown in Figure 1A, the titers of CVS-11 and HEP-Flury in BV2 cells peaked at 12 hpi and then gradually decreased over time. As shown in Figures 1B,C, it was demonstrated that the gRNA amplification and structural protein expression can be carried out normally in BV2 cells. The gRNA gradually increased over time after 12 hpi, and the same result of structural protein expression was observed. Overall, these results suggest that the assembly of viral particles of RABV was inhibited in BV2 cells.

### RABV Infection Leads to the Accumulation of Autophagosomes in BV2 Cells

**Figure 2:**
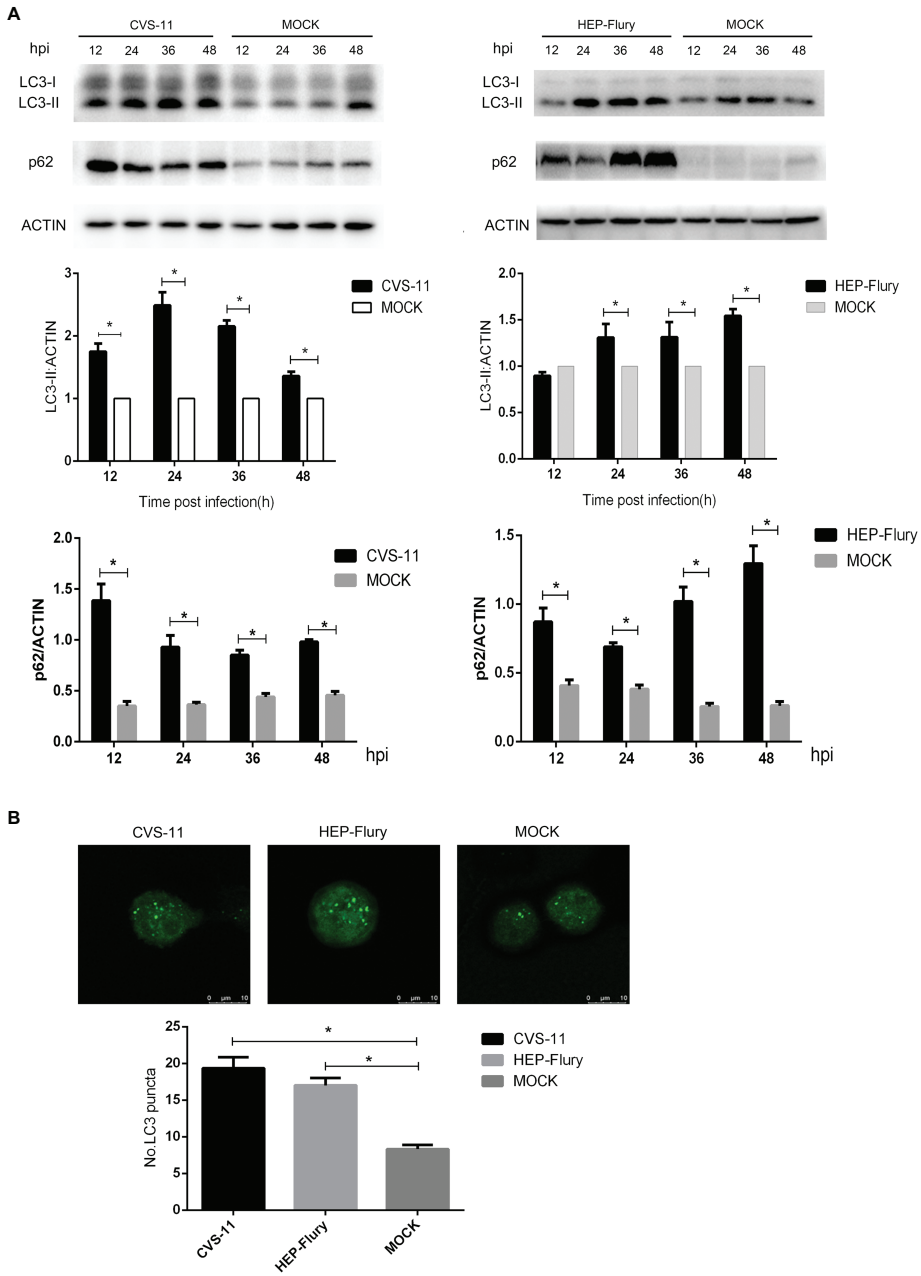
Autophagy induced by RABV in BV2 cells. **(A)** Western blotting to analyze the expression of LC3 and p62 protein. BV2 cells were, respectively, infected with CVS-11 at an MOI of 10 or HEP-Flury at an MOI of 3. Cell lysates were harvested at 12, 24, 36, and 48 hpi. Western blot analysis was used to assess the relative expression levels of LC3-II, p62, and β-actin. Data are represented as mean ± SD, *n* = 3. Asterisks indicate significant differences between the groups calculated using a Student’s *t*-test (^*^p < 0.05). **(B)** LC3 dots in BV2 under laser confocal fluorescence microscopy. BV2 cells were transfected with plasmids and then infected with CVS-11 or HEP-Flury at an MOI of 3. The fluorescent dots in BV2 cells were directly observed at 12 hpi. The average number of LC3 dots was determined from at least 50 cells in each group. Data are represented as mean ± SD of three independent experiments. Asterisks indicate significant differences between the groups calculated using a Student’s *t*-test (^*^p < 0.05).

To verify whether RABV induces autophagy in BV2 cells, LC3-II and p62 expression was investigated in BV2 cells. First, we evaluated the conversion of LC3-I to LC3-II *via* immunoblotting. LC3-II was found to be highly expressed in BV2 cells infected with RABV. As shown in Figure 2A, LC3-II expression in BV2 cells infected with CVS-11 or HEP-Flury was higher than that in non-infected cells after 24 hpi. The autophagy cargo protein p62, which is one of the autophagy biomarkers, was increased significantly in cells infected with RABV. The BV2 cells were transfected with fluorescent-tagged LC3 plasmid and subsequently infected with CVS-11 or HEP-Flury. As shown in Figure 2B, the number of EGFP dots on visualizing BV2 cells infected with CVS-11 or HEP-Flury was significantly higher than that on visualizing mock infection. Overall, these results suggest that RABV can induce autophagy in BV2 cells.

### RABV Infection Leads to the Accumulation of Autophagy Cargo Protein in Autophagy Inhibitor-Treated BV2 Cells

**Figure 3:**
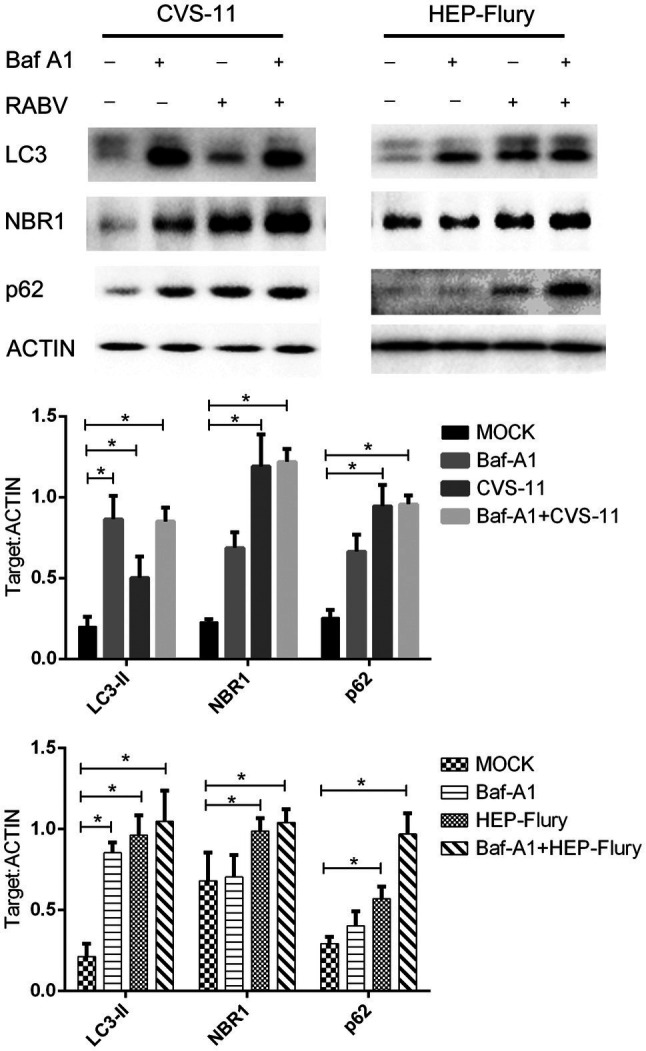
Western blotting was used to analyze the expression level of p62, NBR1, and LC3. BV2 cells were incubated with 100 nM Bafilomycin A1 for 2 h and then were infected with CVS-11 or HEP-Flury at an MOI of 3. Cell lysates were harvested at indicated time points and Western blotting was performed using anti-p62, anti-NBR1, and anti-LC3 antibodies. The results present the relative expression levels of p62, NBR1, LC3-II, and β-actin visualized using the ImageJ software. Data are shown as mean ± SD, *n* = 3. Asterisks indicate significant differences between the groups calculated using a Student’s *t*-test (^*^p < 0.05).

Mature autophagosomes fuse with lysosomes to be degraded, and the autophagic degradation activity is closely associated with the occurrence of many diseases (Zhang et al., 2013). To monitor the further accumulation or degradation of autophagosomes induced by RABV in BV2 cells, we used Bafilomycin A1 (Baf-A1; an autophagy inhibitor) to treat cells and analyzed LC3, p62, and NBR1 protein expression after infected RABV by Western Blot. As shown in Figure 3, cells infected with RABV had significantly higher expression levels of LC3-II, p62, and NBR1 (an autophagy cargo protein) than cells with mock infection. The expression of LC3-II, p62, and NBR1 slightly increased in Baf-A1-treated cells after infection with HEP-Flury compared to uninfected, indicating that autophagy was further induced after viral infection, while autophagy substrates were also accumulating. However, in CVS-11 group, the expression of LC3-II did not increase in Baf-A1 treated cells after viral infection, and at the same time, the expression of p62 and NBR1 was slightly increased, demonstrating that CVS-11 did not induce treated cells autophagy, and caused autophagy cargo protein accumulation. To summarize the results, RABV infection leads to the accumulation of autophagy cargo protein in autophagy-inhibited BV2 cells.

### RABV Infection Inhibits the Fusion of Autophagosomes With Lysosomes

**Figure 4:**
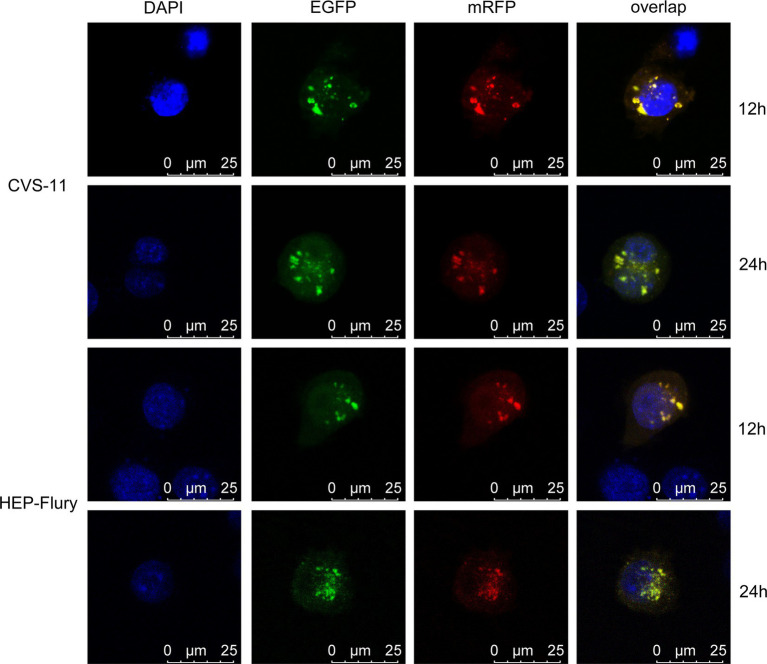
To monitor the fusion of autophagosomes with lysosomes, BV2 cells were transfected with mRFP-EGFP-LC3 plasmids and then, respectively, infected with CVS-11 or HEP-Flury at an MOI of 3. The fluorescent dots in BV2 cells were analyzed directly at 12 and 24 hpi by confocal microscopy.

BV2 cells were transfected with mRFP-EGFP-LC3 plasmid and then infected with RABV. The fusion of autophagosomes with lysosomes was monitored at 12 and 24 hpi by observing the co-localization of red and green fluorescence in the cells. As shown in Figure 4, the red dots and green dots in the cells are overlapped (yellow dots), and no isolated red spots are visible. Because of the acid resistance of the red fluorescent protein, it only shows red dots if the autophagosomes are fused with lysosomes. Therefore, RABV infection inhibits the fusion of autophagosomes with lysosomes.

### Different Multiplicities of RABV Infection Induce Different Degree of Autophagy in Cells

**Figure 5:**
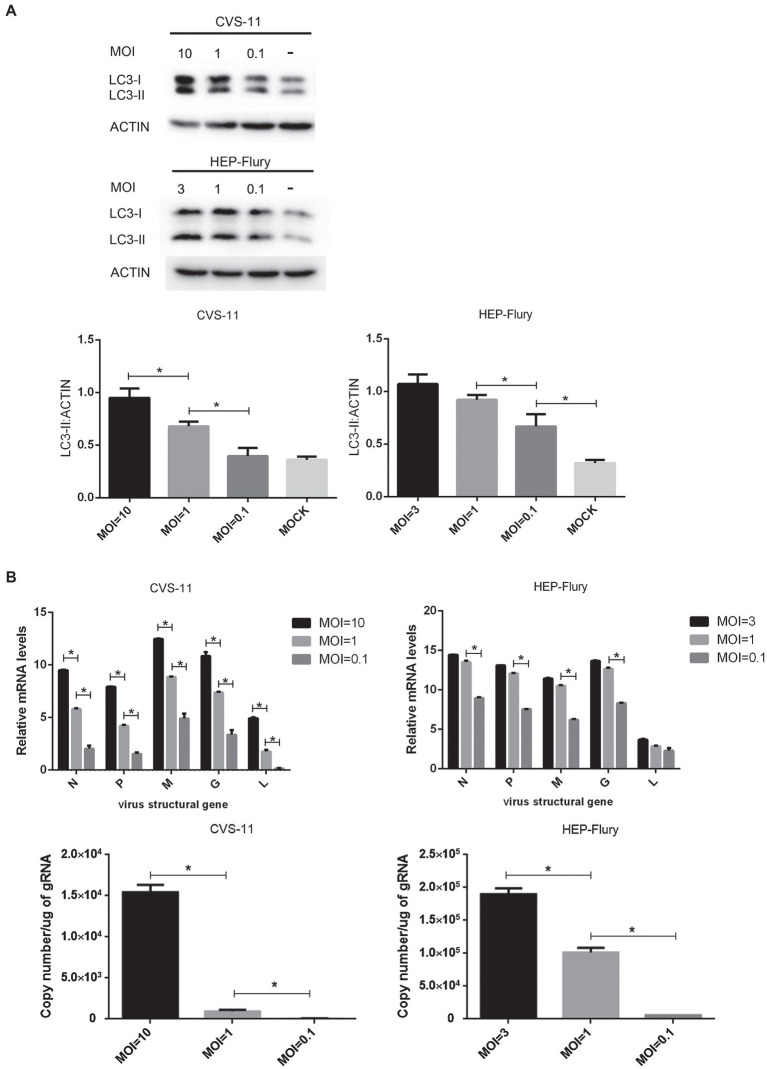
Investigation of the relationship between multiplicity of infection and autophagy. **(A)** Immunoblots are used to detect the level of LC3 expression. BV2 cells were, respectively, infected with CVS-11 or HEP-Flury at different MOIs. Cell lysates were harvested at 6 (CVS-11) and 12 (HEP-Flury) hpi for Western blot analysis. The results present the relative expression levels of LC3-II and β-actin visualized using the ImageJ software. Data are shown as mean ± SD, *n* = 3. Asterisks indicate significant differences between the groups calculated using a Student’s *t*-test (^*^p < 0.05). **(B)** Detection of RABV gRNA and mRNA syntheses by RT-qPCR. BV2 cells were, respectively, infected with CVS-11 or HEP-Flury at different MOIs. Cells were harvested at 6 (CVS-11) and 12 (HEP-Flury) hpi. The levels of gRNA, N mRNA, P mRNA, M mRNA, G mRNA, and L mRNA in cells were determined by RT-qPCR using a CFX connect real-time system. The copy number of gRNA was evaluated and standardized by RABV genome plasmid, and the relative transcription level of mRNA was normalized by GAPDH. Data are presented as mean ± SD, *n* = 3. Asterisks indicate significant differences between the groups calculated using a Student’s *t*-test (^*^p < 0.05).

To explore the relationship between viral concentration and the degree of autophagy, BV2 cells were infected with RABV at various MOIs and the expression of LC3-II was determined by Western blotting. gRNA and mRNA levels were determined by RT-qPCR. As shown in Figure 5, a high multiplicity of CVS-11 or HEP-Flury infection induced a higher level of LC3-II expression than a low MOI at a given time point (Figure 5A). Simultaneously, higher levels of gRNA and mRNA ratios were observed in cells with a higher MOI (Figure 5B), which indicated that viral nucleic acid replication and protein synthesis were more efficient. Overall, these results indicated that the larger the viral concentration in cells, the higher degree the autophagy induced, which suggests that autophagy is positively correlated with viral load and protein synthesis.

## Discussion

The Japanese encephalitis virus has previously been shown to multiply and produce progeny viruses in BV2 cells (Thongtan et al., 2010). In this study, we confirmed that the production of virus progenies of CVS-11 and HEP-Flury strains was inhibited in BV2 cells, but the gRNA amplification and structural protein expression can be carried out normally. It made BV2 cell line a promising model for investigating the relationship between RABV-induced autophagy and multiplicities of infection. To the best of our knowledge, this is the first study indicating that autophagy induced by RABV has no relation with the pathogenicity of the virus but is rather related to the multiplicities of infection. We further plan to investigate the reasons for this failure to produce viral progeny in our subsequent research.

In our study, on the accumulation of autophagy cargo protein, the results showed that CVS-11 did not induce autophagy in Baf-A1-treated cells. The induction of autophagy was not a superimposed process, and the autophagy induced by CVS-11 was not strong. Therefore, compared to drug treatment, the autophagy induced by infection was negligible. Here, we guess that if we increase the amount of virus inoculation to increase the induced autophagy, CVS-11 will induce autophagy in Baf-A1-treated cells, and autophagy cargo protein will accumulate. If this is the case, then we can conclude that CVS-11 induces incomplete autophagy in BV2 cells.

Our previous study has reported that RABV induces complete autophagy in SK cells, but incomplete autophagy in NA cells (Peng et al., 2016). Therefore, the induction of incomplete or complete autophagy by RABV is cell line dependent. p62, an autophagy receptor protein, mediates the combination of autophagy cargo and autophagosomes, to ensure selective clearing of toxic protein aggregates and injured organelles *via* autophagy. Increasing evidence supports the belief that p62 is associated with the occurrence of neurodegenerative and immune diseases (Lim et al., 2015). However, whether the increased accumulation of p62 caused by RABV in BV2 cells contributes to the process of rabies is unclear and needs further work.

Autophagy can degrade misfolded protein and damaged organelles in cells, and subsequently can provide energy, amino acids, fatty acid, and other nutrients to the cells (Yang et al., 2019). If this process cannot be conducted completely, it results in cell death (Lumkwana et al., 2017). Many human diseases, including cancer, cardiovascular diseases, immunological diseases, and neurodegenerative diseases, are associated with impaired autophagy flux (Zhang et al., 2013). In addition to these, autophagic flux also plays an important role in the viral life cycle. HCV inhibits the maturation of autophagosomes, creating an environment more conducive for its replication (Wang et al., 2015). Dengue virus activates autophagy in the early period of infection to support viral replication, while in the later period, autophagy vesicle accumulation is more conducive to viral replication (Metz et al., 2015). There are many factors that affect autophagic flux, such as ER stress, oxidative stress, and aging (Yin et al., 2017). Studies have shown that various reasons can cause damage to autophagic flux: incompletely formed autophagy vesicles resulting from dysfunction; intracellular dynamin or tubulin protein interference to disrupt the fusion of autophagosomes with lysosomes; and destruction of the acidic environment of the lysosomes or reduced proteolytic enzyme activity (Kimura et al., 2008; Zhong et al., 2009; Nixon, 2013). Our results indicate that RABV can induce autophagy in BV2 cells, although it is incomplete. There are no studies explaining how RABV prevents the degradation of autophagic vesicles, but it is suspected that the blockage of autophagic flux must play a key role in the viral life cycle, which provides a direction for subsequent research.

Our previous study found that different recombinant RABV strains induced different levels of autophagy after infection with the same multiplicities (Peng et al., 2016). Another study also found that different RABV strains induced different levels of autophagosome accumulation in cells (Li et al., 2017). Based on these previous findings, we explored the relationship between autophagy and multiplicities of infection in this study. At the initial stage of autophagy, the greater the viral concentration in cells, the stronger the autophagy induced, indicating that the level of autophagy induced by RABV is associated with the viral load. The proof of this viewpoint can explain some problems encountered in our experiments. For example, we found that when an attenuated strain replaces the M gene of the virulent strain, the recombinant virus can induce autophagy earlier than the parent virus (Peng et al., 2016). By testing the viral load in the cells, we found that the recombinant strain with the replaced M gene had a higher viral load and higher mRNA synthesis in the cell than the parent strain (Yang et al., 2014). Furthermore, when cells are inoculated with RABV at a low MOI, autophagy often appears to be induced later than when cells are inoculated with RABV at a high MOI, in such case sometimes autophagy cannot even be detected. Therefore, we believe that RABV-induced autophagy is related to viral load in cells, which was caused by multiplicities of infection. The original goal of this article was to study the relationship between viral replication and autophagy, but unfortunately, there is not enough evidence to support this issue in the research. Of course, we will continue to study it.

## Conclusion

In conclusion, we proved that RABV can induce autophagy in BV2 cells and that the autophagy is associated with the viral load in the cell. Our findings provide a basis for further exploring the relationship between autophagy and viral infection.

## Data Availability Statement

The original contributions presented in the study are included in the article/supplementary material, further inquiries can be directed to the corresponding authors.

## Author Contributions

XG, JLu, and YW contributed to the study design. YW, HH, JLi, LC, and YK performed the research. YW, ZL, RF, BZ, JLu, and YL contributed to the data analysis. YW wrote the original draft of the manuscript. XG and JLu reviewed and edited the manuscript. All authors contributed to the article and approved the submitted version.

### Conflict of Interest

The authors declare that the research was conducted in the absence of any commercial or financial relationships that could be construed as a potential conflict of interest.
